# Electrical Stimulation of Denervated Muscle: A Narrative Review

**DOI:** 10.1111/aor.70076

**Published:** 2025-12-26

**Authors:** Linshan Chu, Jonathan C. Jarvis, Brian J. Andrews, James J. FitzGerald

**Affiliations:** ^1^ Nuffield Department of Surgical Sciences University of Oxford Oxford UK; ^2^ Faculty of Health, Innovation, Technology and Science Liverpool John Moores University Liverpool UK

**Keywords:** artificial reinnervation, denervated muscle stimulation, direct muscle stimulation, electrical stimulation, foot drop, functional electrical stimulation, indirect muscle stimulation, muscle transfer, neuronal grafts

## Abstract

**Background:**

Electrical stimulation is commonly employed for activation of paralyzed muscles in patients with neurological diseases and injuries. However, there are differences in the treatment approaches that are possible for upper motor neuron and lower motor neuron injuries.

**Methods:**

This narrative review synthesizes findings from preclinical studies and clinical reports published over the past decades. Key topics include stimulation parameters, muscle physiology under denervation, and outcomes of invasive and noninvasive interventions. The selection of sources was based on their relevance to denervated muscle stimulation in both experimental and therapeutic contexts.

**Results:**

This review critically examined the physiological and therapeutic differences between indirect and direct stimulation of muscles in upper motor neuron and lower motor neuron injury situations. It then focused on the much less well‐established field of stimulation of denervated muscle, where there remains a pressing need for new clinical approaches. We explained the rationale for stimulating denervated muscles and the practical difficulties encountered in doing so, describing the use of both invasive and noninvasive devices in animal experiments and clinical trials. We then discussed related research using artificial reinnervation for denervated muscle stimulation and suggested directions for future exploration in this dynamic field.

**Conclusion:**

Stimulation of denervated muscle remains a promising but underdeveloped area. Electrical stimulation of denervated muscle can preserve muscle mass and potentially restore function. However, its clinical adoption has been limited by the exceptionally high stimulation thresholds required, which are approximately one thousand times higher than those for indirect muscle stimulation via intact lower motor neurons. These demands lead to significant challenges including discomfort, limited specificity due to the need for large electrodes, and the risk of tissue damage. Artificial reinnervation may offer a promising solution by enabling the use of conventional low‐energy stimulation techniques. Additionally, the application of stimulation in free muscle transfers may further expand therapeutic options in this area.

## Graphical abstract

This review explores existing strategies for stimulating denervated muscle following lower motor neuron injury, including direct electrical stimulation of the muscle and artificial reinnervation to enable more comfortable and efficient indirect functional muscle stimulation. It highlights current challenges and outlines potential future directions aimed at developing more effective and patient‐centered approaches to restore muscle function after denervation.

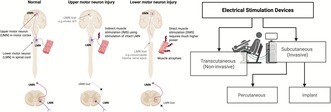

## 1 Motor Neuron Injuries

**FIGURE 1:**
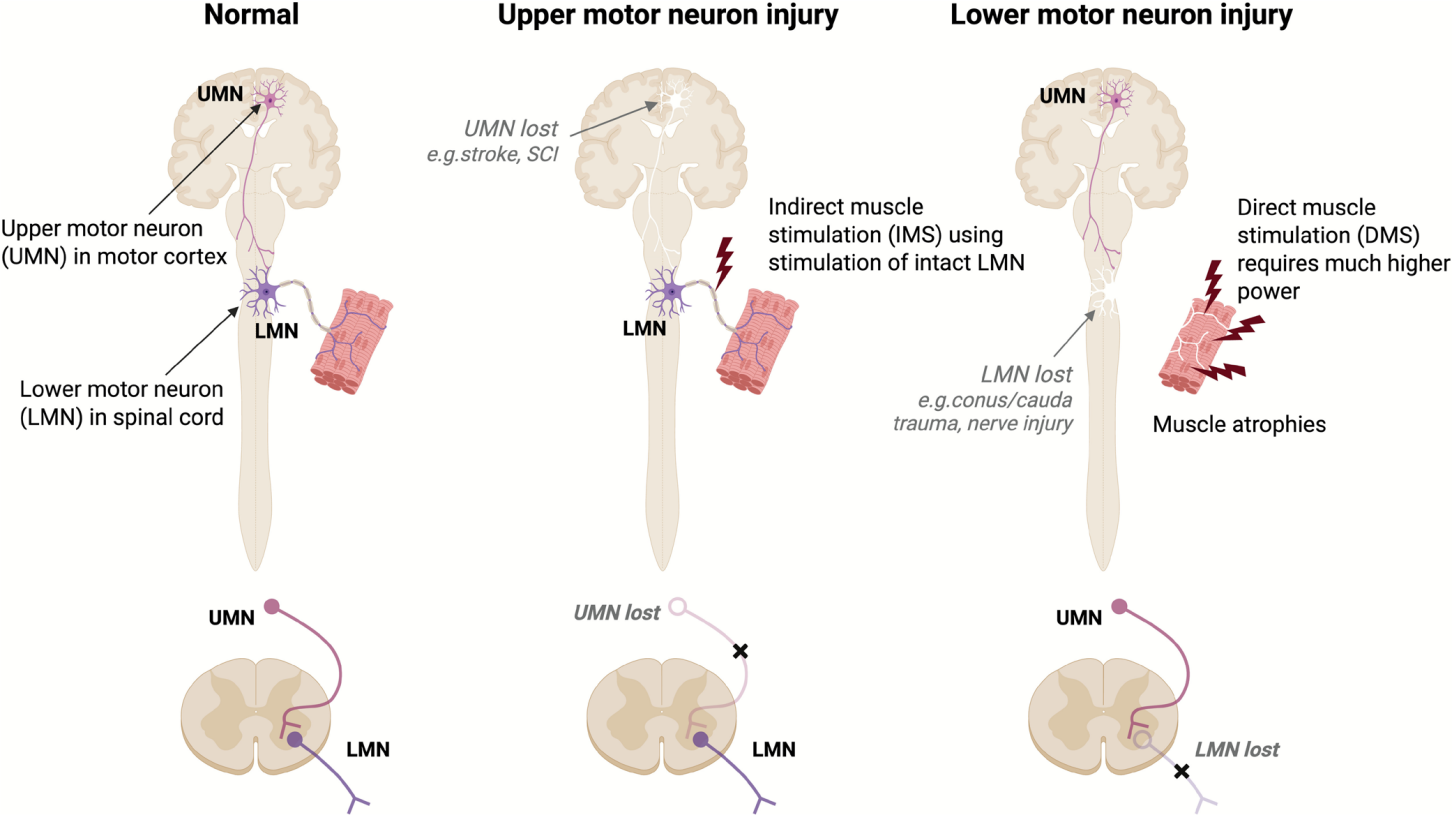
Patterns of motor neuron injury. (a) Normal arrangement showing upper motor neuron (UMN) and lower motor neuron (LMN). (b) UMN injury—LMN still intact and can be used to activate muscle. (c) LMN injury—activation of muscle by nerve stimulation is not possible. [Color figure can be viewed at wileyonlinelibrary.com]

There is a two‐neuron pathway from brain to muscle that is responsible for translating movement intention into muscle contraction, and consequently, actual movement (Figure 1a). The upper motor neuron (UMN) has its cell body in the primary motor cortex, the brain area responsible for voluntary movement. Its axon runs within the corticospinal tract, where it synapses with the lower motor neuron (LMN) in the anterior horn of the gray matter. The LMN then sends its axon from the spinal cord to the muscle via peripheral nerves. Many such arrangements operate in parallel (typically, several hundred LMN axons run to each muscle). Injury at either the UMN or LMN level leads to muscle paralysis to differing degrees, but the location of the damage determines the resulting clinical syndrome.

UMN injuries (Figure 1b) result from damage to descending motor pathways, primarily the corticospinal tract, which runs from the motor cortex through the spinal cord. Common UMN lesion causes include stroke (cortical or subcortical), spinal cord injury (SCI), and demyelinating disorders like multiple sclerosis (MS). In an UMN lesion, lower motor neurons remain anatomically intact, but descending control (mainly from corticospinal and other supraspinal tracts) is lost or impaired. LMNs without input from above tend to become hyperactive, remaining part of intact signal reflex arcs that become exaggerated. Thus, although the muscle is paralyzed (unable to be voluntarily controlled), it is stiff, and reflexes are abnormally brisk; this is known as spastic paralysis.

LMN injuries (Figure 1c) result from damage to the spinal cord at the LMN level, spinal nerve roots, or peripheral nerves. After a peripheral nerve injury, the part of the axon distal to the injury site undergoes Wallerian degeneration; muscles that lose innervation quickly atrophy and develop fasciculations, and lose reflexes. Some residual activity and reflexes may persist in partial denervation cases. Very proximal injuries, close to or within the spinal cord, can result in the death of the entire motor neuron. However, if some rootlets are spared, partial function or reflexes may remain. In some chronic cases, fibrillations (spontaneous activity of denervated muscle fibers) may also develop [1], though these are not visible clinically. If all its motor axons are damaged, the muscle loses its axonal input, and all activity—both voluntary contraction and reflex‐mediated contraction—is absent. The muscle generates no force, a condition known as flaccid paralysis. Muscle denervation, as seen after LMN injury, also causes pronounced muscle atrophy, with a loss of muscle fiber diameter and overall muscle mass due to the absence of muscle contraction in response to electrical activation and the loss of chemical trophic factors derived from the nerve. Eventually, denervated muscle undergoes irreversible changes, including the transformation of muscle into adipose and fibrous tissue [2].

Electrical stimulation (ES) is an important clinical intervention for treating nerve injuries. In cases of UMN injury, the LMN remains intact; therefore, stimulation can be applied at the LMN level to induce muscle contractions, a technique commonly referred to as functional electrical stimulation (FES). In contrast, when the injury involves the LMN, stimulation must be applied directly to the muscle, a method known as direct muscle stimulation (DMS). In this review, we will further discuss the distinctions between these stimulation modalities in subsequent sections.

In many neurological injuries, muscles may be partially denervated due to a combination of upper and lower motor neuron involvement. This is particularly challenging in tetraplegic patients undergoing hand and arm reconstructive surgery, where accurately assessing the degree of denervation is critical for planning interventions such as nerve transfers [3]. Nerve and tendon transfers have become increasingly prominent in current reconstructive strategies and offer potential functional restoration when applied appropriately [4, 5].

## 2 Indirect Muscle Stimulation

ES has been widely investigated as a means of rehabilitating paralyzed muscles, restoring movement and producing a less wasted appearance [6, 7]. Activating paralyzed muscles can also provide cardiovascular benefits [8] and aid in the prevention of pressure injuries [9]. A summary of different types of stimulation is shown in Table 1.

**TABLE 1:** Different types of electrical stimulations.

	Types of electrical stimulation (ES) for neurological damage and disorders		Aim	Principle
Indirect muscle stimulation (IMS)	Transcutaneous electrical nerve stimulation (TENS)		Pain relief	Stimulation of nerves to disrupt pain signal transmission to the brain
	Neuromuscular electrical stimulation (NMES)	Conventional Functional electrical stimulation (FES)	Functional restoration	Stimulation of motor neuron to induce coordinated muscle contraction
		Electromyogram triggered stimulation (ETS)	Stimulation of muscles that still receive some neuronal signals when patients conduct voluntary movement or have intention to move, due to incomplete injury	EMG detects the signal when patient contracts muscle, then the device will send stimulation pulse
		Reciprocal electromyogram triggered stimulation (RETS)	Relieve the muscle stiffness and spasticity	Triggers on relaxation of hypertonic antagonists to help achieve muscle relaxation
Direct muscle stimulation (DMS)			Maintain muscle structure and function after LMN injuries (denervation) Some research uses high power FES to directly stimulate the muscle in order to achieve functional restoration	Muscle fiber membrane depolarisation

ES can activate muscle contraction either by depolarising the axons of LMNs (i.e., peripheral motor nerves), which activate muscle fibers via neuromuscular transmission, or by directly depolarising the muscle fiber membrane itself (direct muscle stimulation or long pulse stimulation). FES is a specific type of ES that focuses on achieving functional movement outcomes, rather than pain relief and other targets included in the broad ES category (Table 1). In practice, a vast majority of clinical applications of FES operate by stimulating intact motor nerves; this type of FES will be referred to as “conventional FES”. In general, stimulation of axons partway along their length will generate action potentials that propagate in both directions from the point of stimulation, rather than in one direction from the cell body to the axonal terminal as they naturally do. The signal propagating retrogradely (known as antidromic propagation) to the neuron cell body is not known to have any deleterious effect [10]. Activating muscle fibers in this way is power‐efficient because nerve tissue is excitable at much lower current than muscle. In conventional FES applications, where the LMN is undamaged, surface hydrogel electrodes are applied over the location of nerve trunks running to a muscle. In certain conventional FES devices currently used in clinical practice, stimulation electrodes are positioned on the skin overlying the target muscle [11, 12]. This placement can easily be mistaken for direct muscle stimulation, as the electrodes appear to be applied to the same region. However, these systems primarily act by stimulating nerve endings within the muscle or as they enter it, rather than directly stimulating the muscle fibers themselves, as illustrated in Figure 1b. In rare cases, FES is applied for direct muscle stimulation to treat long‐term denervated patients who have completely lost their upper motor neuron connections. In such situations, stimulation must be delivered directly to the muscle tissue. This specific application of FES will be introduced in greater detail in Section 3.

A well‐established use of FES is in the treatment of foot drop resulting from UMN injury. Foot drop is caused by the loss of dorsiflexion of the foot (i.e., the ability to lift the forefoot upwards at the ankle joint) due to weakness of the tibialis anterior, extensor hallucis longus, and extensor digitorum longus muscles or the corresponding nerves. This leads to dragging of the foot and potentially tripping over the toes [13, 14]. Patients with foot drop often develop a compensatory ‘steppage’ gait, lifting the affected foot well off the floor during the swing phase. Foot drop can be caused by either UMN or LMN injuries. In cases of UMN injuries, such as multiple sclerosis and stroke, foot flexion and extension need to be externally stimulated for the patients to conduct both the foot‐lift and the push‐off move, in order to achieve the most physiologically correct gait. FES targeting the peroneal nerve has shown positive results in clinical trials for increasing muscle plasticity, knee flexibility, and ankle control [15, 16, 17, 18, 19]. Foot drop due to LMN lesions, such as injuries to the sciatic or peroneal nerves, is not amenable to conventional FES at present.

Up to one‐third of patients with thoracolumbar spinal cord injury (SCI) have injuries at the level of the tip of the cord (conus) or the nerves immediately below it (cauda equina) [20], which produces an LMN‐type injury. These patients, along with all patients whose muscle paralysis is due to major peripheral nerve trauma, such as brachial plexus injury or sciatic nerve injury, are presently unsuitable for conventional FES. However, in cases of LMN injury leading to muscle denervation and loss of function, artificial reinnervation represents a potential approach to enable indirect stimulation of the affected muscle. This strategy will be discussed in greater detail in Section 4.

## 3 Direct Muscle Stimulation

For denervated muscle, stimulation to achieve the beneficial effects of active contraction must target the muscle fibers directly. Sufficiently depolarizing the muscle cell membrane directly requires large amounts of energy [21], nearly three orders of magnitude more than activation via LMN axons [22], and this presents several issues. The challenges of finding the right pulse shape using long pulse widths in order to avoid possible discomfort or keep it at an acceptable level for the patient during stimulation are great. The high current amplitudes and longer pulse widths required can cause discomfort to patients who have intact sensation in the area where stimulation is applied. The total power applied to tissue is of concern (particularly in those who do not have intact sensation) and can approach or exceed safety limits that exist to prevent tissue damage. According to EU regulations, the highest power that can be safely applied to the human body during ES therapy is 300 mJ per impulse [23]. Furthermore, the threshold in terms of current density above which there is a risk of tissue damage has been widely considered to be 30 μC/cm^2^ for macroelectrodes [24, 25]. Beyond the risk of thermal injury, high levels of charge transfer may induce electrolytic reactions at the electrode surface, this process including platinum dissolution and iridium oxide delamination potentially generating harmful chemical byproducts that contribute to tissue damage, though the exact effect on human tissue has not been systematically verified [26]. Therefore, the electrochemical safety limit, governed by current density thresholds, is also a key consideration in the safe application of ES therapies [27, 28].

Delivering high energy requires larger electrodes to distribute the stimulation current for reasons of comfort and avoidance of tissue damage. The use of large‐size electrodes can reduce stimulation accuracy and result in undesired stimulation of adjacent muscles [10]. A further problem is the unevenness of muscle activation. The electric field will be highest closest to the electrode [29], and thus direct muscle stimulation may fail to reach deeper parts of the muscle, in contrast to FES via the nerve, which will activate the muscle more evenly as the axons arborise within it. These limitations have largely prevented DMS from translating to clinical use. Despite the difficulties described above, there are good reasons for developing methods to stimulate denervated muscles. The two main aims are to reduce muscle atrophy and to recover volitional function [10, 11]. Preserving as much muscle mass as possible is particularly important in cases of peripheral nerve injury where there has been nerve repair and there is a realistic prospect of axonal regeneration to the neuromuscular junction, and the goal here is to maintain muscle condition while awaiting potential reinnervation. Reducing atrophy may also prevent pressure injury formation and have cosmetic benefits [30, 31, 32]. The benefits of applying ES to denervated muscle additionally include the promotion of cardiovascular function, if a sufficient mass of muscle can be activated [33]. For volitional functional recovery, the aim is to restore muscle force and improve muscle fatigue resistance. This approach aims to assist patients in muscle contraction, enabling them to complete tasks such as walking, reaching, and grasping [10, 34, 35].

### 3.1 Invasive and Noninvasive Devices

Previous studies stimulating denervated muscle have used both subcutaneous stimulation (invasive) and transcutaneous stimulation (noninvasive, i.e., stimulation applied at the skin surface). Invasive stimulation can be further categorized into percutaneous—that is, externally powered, with wires passing through the skin to the target—or fully implanted. The advantages and disadvantages of each are similar to conventional FES. Table 2 provides a comparison between noninvasive and invasive devices.

**TABLE 2:** Noninvasive and invasive devices.

Type of devices		Advantages	Disadvantages
Noninvasive	Transcutaneous	No implant surgery needed Low cost No infection risk	Cannot reach the deep muscle Large power needed Need to don and doff regularly
Invasive (subcutaneous)	Percutaneous	Can reach the deeper muscle groups Suitable for short‐term stimulation requirements	Infection risk Special care needed
	Implant	Continuous stimulation without donning and doffing Can reach the deeper muscle groups Less stimulation intensity	Expensive Surgery needed Infection risk

Transcutaneous electrodes are applied to patients' skin surfaces and stimulate the muscle underneath. The advantages include lower cost, no surgeries, and no infection risk or requirement for special care in clinic. Patients who use wearable stimulation devices can manage them at home after the necessary instruction [36]. However, as the skin presents high impedance, larger power is needed than with implanted electrodes, and for patients with denervated muscles but an intact sensory system, it can be very uncomfortable. Also, to reduce the focal current density while maintaining the current amplitude, the electrode is always larger than subcutaneous electrodes [37]. This is unavoidable in DMS compared to indirect muscle stimulation (IMS), where smaller power is used, and research shows that selective IMS in forearm to induce individual finger contraction and extensions is achievable [38]. The compromise between high power, stimulation comfortability, and the size of electrodes will result in a lack of muscle selectivity and can lead to stimulation of unwanted muscle fibers, limiting use in deep or fine muscle fiber stimulation. For long‐term usage, an additional drawback is the need to don and doff regularly and to carry a bulky stimulation device. This method requires more time for treatment and more effort on patient education but may still be useful, for instance, in rehabilitation after LMN injuries to prevent muscle atrophy while reinnervation is awaited [22, 39, 40].

Subcutaneous stimulation is more efficient and requires less power compared to using surface electrodes, and it may be more comfortable for patients during stimulation. Percutaneous stimulation—where wire electrodes are inserted into the skin to reach the target muscle—provides the ability to reach deep muscle groups without surgery. However, it has a high and continuous risk of causing infections, and patients need special care in clinic [41, 42, 43]. Because of this, it is generally a temporary arrangement and may be a good selection for short‐term stimulation.

Fully implanted systems offer the greatest convenience, as they obviate the need for donning and doffing, eliminate reliance on external hardware, and require minimal day‐to‐day maintenance. Implanted electrodes are best suited for chronic applications where stable, long‐term stimulation is desired. However, their use needs a surgical implantation procedure, which increases the cost and carries a short‐term infection risk at the time of implantation.

### 3.2 Animal Studies

The vast majority of animal models have used subcutaneous electrodes, either percutaneous or implanted.

Multiple studies, including several dating back to the 1940s, have shown that stimulation of denervated muscles retards their atrophy [44, 45, 46, 47, 48] (Table 3). These have been conducted mainly in rats [44, 45] and rabbits [46, 48]. Stimulation applied to the rabbit extensor digitorum longus (EDL) muscle (at 10–12 Hz) has also been shown to reduce the shift in twitch/tetanus ratio and the prolongation of contraction and relaxation that are seen after denervation [30]. In a study of DMS of the denervated EDL muscle in rats, performed to quantify the amount of stimulation needed to retard muscle atrophy [49], it was found that contractile force could be preserved with between 200 and 800 tetanic contractions per day, with equal interval resting time in between.

**TABLE 3:** Summary of experimental and clinical studies on ES in denervated muscle models.

Species	Model	Stimulation parameters
Animal	Rats (soleus, exterior digitorium longus (EDL) in experiments) [44]	Stimulated denervated rat soleus with brief 100 Hz trains vs. long 10 Hz trains for weeks
	Rats (tibialis anterior muscle denervation) [45]	Tetanic stimulation of 300 ms duration, 60 Hz, 4 stimulations per minute, followed by a 2‐min rest period. Pulse duration 1 ms
	Rabbits [46]	20 min galvanic exercise, current strength 4–6 mA, 8 mm and 3 mm copper electrodes, applied to denervated (short term 37 days and long term 67–150 days)/reinnervated rabbit muscle
	Rabbits [47]	Twice daily, 40–60 tetani of 1–2 s duration, with appropriate resting periods.
	Rats (EDL denervated rat muscle) [49]	Stimulated denervated EDL to produce 25–5000 contractions/day (tested); found 200–800 tetanic contractions/day preserved force and mass
	Rats (denervated soleus and EDL) [50]	Wire‐like Teflon‐coated subcutaneous electrodes; compared 10 Hz vs. 100 Hz DMS applied 2–3 weeks post‐denervation
	Rabbits (tibialis anterior muscle, implanted stimulators in rabbits) [51]	Implantable microcontroller stimulator, RF programming, stainless‐steel foil electrodes; devices functional up to 36 weeks. 100 ms per phase, and 10 bipolar constant‐current pulses, then adopt stepwise protocol. For 10–36 weeks stimulation, 1–5 h/day stimulation with one of different patterns.
	Rabbits (EDL denervation model) [52]	Five stimulation patterns delivering 24 000–480 000 impulses/day applied for 6–10 weeks; high‐intensity paradigms tested
	Rabbits (10‐week denervation) [53]	20 ms duration per phase, 4 mA amplitude, 20 Hz duty cycle of 1 s on, 2 s off; a total of 24 000 impulses per day
	Rabbits (up to 1 year denervation) [54]	“Supramaximal level” stimulation pulse, 0.2‐ms and 10‐ms pulse widths, used same device as [51]
	Canines (posterior cricoarytenoid muscle) [55]	36‐electrode planar array implant; chronic stimulation to denervated muscle prior to reinnervation 1 s, 30 pps, biphasic pulse train composed of 1‐ms pulses 2–6 mA in amplitude, repeat every 10 s
	Rats (sciatic nerve models) [56]	Brief ES: Supramaximal pulses (100 μs; 3 V), continuous 20 Hz train, applied to nerve proximal to repair site
	Rats (femoral nerves) [57]	Continuous 20 Hz stimulation (100 μs, 3–5 V)
Human	Human (conus‐level SCI, denervated lower limbs, 9–18 months) [34]	Surface silicone‐graphite electrodes (≈200 cm^2^); start 120–150 ms pulses at 1–2 Hz, progressed to 30–50 ms pulses at 16–25 Hz; training over months to years
	Human (3 patients) [35]	Upgraded multi‐channel stimulators (8 channels), smaller electrodes (~97 cm^2^); variable parameter control via software
	Human (used in Project RISE clinical protocol) [37]	Stimulette den2x: pulse width 1–300 ms, ±80 V amplitude, up to 250 mA; initial long pulses (120–150 ms) with stepwise shortening
	Human (SCI patients, *n* ≈ 40 in trial) [58, 59, 60, 61]	Stimulette den2x home‐based training (pulse width 1–300 ms; high voltages ±80 V; up to 250 mA); started with long pulses (120–150 ms) then shortened progressively

It is well known that the pattern of stimulation in innervated muscle can affect the fiber type composition of the muscle [62]. Stimulating a fast muscle at a continuous low rate makes it slower, while depriving a slow muscle of the low‐rate stimulation that it normally receives makes it faster. There is some evidence to suggest that there is a similar modulation of some cellular properties of denervated muscle by DMS [44]. Acetylcholinesterase (AChE), which hydrolyses acetylcholine at the neuromuscular junction, is expressed in different forms in normal fast and slow muscle. Lomo's team [50] investigated the effect on AChE expression of stimulating denervated muscle with both 10 Hz and 100 Hz frequencies. They subcutaneously implanted wire‐like Teflon‐coated electrodes in rats' legs and conducted DMS 2–3 weeks after soleus (SOL) (slow muscle) or EDL (fast muscle) denervation. They found that the different stimulation frequencies produced distinct patterns of expression of AChE molecular forms, with 10 Hz stimulation and 100 Hz stimulation respectively evoking expression patterns similar to normal slow and fast muscle.

In 2005, the Medical University of Vienna and the University of Liverpool developed an implantable stimulator for denervated muscle [51]. The microcontroller‐based device is programmed through a bidirectional radiofrequency link and has electrodes made of stainless‐steel foils with silicone‐coated rims. 55 devices were implanted intraperitoneally into laboratory rabbits, of which 53 remained functional for periods up to 36 weeks. Using this device, it was possible to noninvasively track changes in excitability of denervated skeletal muscles over time. A fivefold increase in chronaxie was observed in the 2 weeks following denervation.

Ashley et al. used this device to study the influence of stimulus pattern on the effectiveness of denervated muscle stimulation in rabbits [52]. To more closely simulate clinical cases, they delayed the start of stimulation until 10 weeks after the denervation surgery, by which time muscle mass had decreased by 60%. Protocols ranging between 24 000 and 480 000 daily impulses were investigated using various functional recovery indicators. High‐intensity ES reduced muscle atrophy, but the effect was not significantly dependent on stimulation patterns. The investigators reported comparable outcomes in denervated muscle when applying 24 000 impulses per hour per day and when using a pattern with a 20‐fold greater impulse count. Thus, even if pattern affects properties like AChE expression, as described above, this does not appear to translate into differences in muscle mechanics.

It is clear that some consequences of denervation cannot be modified with current means of compensating for lost muscle activation by ES [53, 63]. Compared to innervated muscle, the contraction speed of denervated muscle when stimulated is significantly slower [7], and this characteristic is independent of the stimulation pattern applied [52]. Higher mitochondrial density has been found in denervated muscle [54], but ES of denervated muscle does not produce such a marked increase in resistance to fatigue as it does in innervated muscle [7]. Denervated muscle will return to an atrophied state in a short time if ES treatment is not continued [7].

Finally, there is considerable evidence to suggest that where denervation is due to peripheral nerve injury, ES may also be usefully applied to the nerve at or proximal to the injury site, where it may promote nerve regeneration [55, 56, 57, 64].

### 3.3 Human Trials

In 1999, Kern et al. [34] demonstrated that patients with denervated lower extremity muscles due to conus‐level spinal cord injury, with denervation periods of some 9–18 months, could recover the ability to stand (between parallel bars) using DMS, although this required training for 1–2 years. Stimulation was applied to the denervated muscle using surface electrodes, and the research group had to develop a new stimulator that could produce higher power than the systems available on the market. Large (200 cm^2^) anatomically shaped silicone‐graphite electrodes were placed on the skin over the target thigh muscle. Beginning with very broad (120–150 ms) single pulses at low frequency (1–2 Hz) for short periods, and then gradually decreasing pulse duration and increasing pulse rate and training time, a 4%–6% strength increment per month was achieved, and parameters of 30–50 ms pulse duration and 16–25 Hz frequency to produce fused (tetanic) contractions were reached.

Based on the work of Kern and Mayr in the FES Vienna group, 19 European organizations across 13 countries established a consortium to use FES to develop a new rehabilitation pathway for long‐term flaccid paraplegia patients, aiming to achieve standing, named Project RISE [58, 59]. This project ran from 2001 to 2006. Experiments in rabbits and pigs were followed by a clinical trial in 40 patients with spinal cord injuries [60]. A two‐channel home‐based FES (h‐bFES) stimulator was developed (Stimulette den2x), specifically designed for denervated muscle stimulation and controlled by either a laptop or a user‐friendly digital interface [37]. The stimulator could generate four types of waveforms, including rectangular, bipolar and ramped shapes, with adjustable 1–300 ms pulse width, ±80 V voltage amplitude, and 250 mA current. Patient training started with long‐duration (120–150 ms, 60–75 ms per phase) and high‐intensity (±80 V and ± 250 mA) stimulation and then shifted to shorter pulse durations step by step as muscle activity and sensitivity increased [59]. The results showed the positive effect of DMS‐FES therapy on long‐term denervated muscle by reversing muscle atrophy, maintaining muscle structure, increasing muscle mass and tetanic contractility, and improving skin cushioning [31, 60, 61]. By assisting knee extension, five out of twenty patients achieved the ability to stand up, and three of them could walk for several meters between parallel bars using their upper extremities for support and balance [58].

Hofer et al. [37] proposed the idea of integrating the FES stimulation device into specially designed garments, both to help ensure that the device elements are all applied reproducibly in the correct place and to improve convenience and safety for patients. Several groups are developing cloth and electrode materials to optimize stimulation performance [65, 66].

Although the results of Project RISE were very encouraging, the research group highlighted several significant issues [63], including differing results in excitability and force‐generating capacity obtained from rabbit and human studies, an issue discussed further below. Other areas highlighted for improvement in future work included the optimization of stimulation patterns and parameters to reduce the large power used; while the stimulator appeared safe for use in muscle‐denervated patients, the power required exceeded the maximum power allowed by EU regulations in ES therapies [22].

Hofer et al. [37] first reported the design of a two‐channel stimulator for activating denervated muscles in 2002 [37], noting their intention to develop a four‐channel device to enhance electrically assisted functional training for patients. Currently, commercially available devices for large‐muscle stimulation include the two‐channel RISE stimulator and the four‐channel StiwellPROFES, both incorporating dedicated programs for denervated muscle stimulation. More recently, an eight‐channel stimulator [35] has been introduced, with independent channel control to stimulate eight major muscle groups in the leg. This eight‐channel stimulator is neither currently applied in clinical practice nor commercially available. In their reported device, smaller size electrodes (around 97 cm^2^) were used, with the stimulation parameters controlled by specially designed software on a PC or mobile phone via Bluetooth connection. Three subjects who had been denervated for a year and had profound muscle atrophy were trained for a month. The authors reported that this was easier to administer than previous systems and safe for hospital and home use, with no stimulation‐related cutaneous side effects, and from a baseline of no response to stimulation, contractile activity (as measured by a novel sensor) was elicited in all muscles studied in all the patients. However, this study has a major limitation regarding reproducibility, both in terms of the patient population examined and the methodology employed. More results from dynamometry, longer trial durations, more control group data, and larger sample sizes are required to substantiate the reliability and clinical utility of this type of stimulator.

Here, in Table 3, we provide a comprehensive summary of the denervated models and corresponding stimulation parameters from the studies discussed in this section, organized into *animal* and *human* categories as outlined in 3.2 and 3.3.

### 3.4 Animal Models and Translatability

Several animal species have been used in DMS research, including frogs, rats, rabbits, cats, dogs, and pigs. Stimulation parameters and outcomes can vary greatly depending on the species used as experimental subjects, and animal models must be carefully selected according to the research goals. As pointed out by Salmons and Jarvis [7], muscle degeneration begins relatively soon (several months) after denervation surgery in rats [67, 68], but rabbits exhibit nondegenerative atrophy that is stable for at least 51 weeks post injury, which better reflects the situation in humans [54]. Additionally, attention should be paid to the post‐surgery time of denervation in animal models, as most clinical cases involve patients who have experienced muscle denervation for months to years. In modeling these cases, stimulation cannot be applied immediately after denervation surgery but must be delayed to obtain more reliable results [7]. A further limitation of animal models is that stimulation durations are limited due to the animals' lifespan, making long‐term training infeasible [7]. Partly as a consequence of these factors, even though a considerable number of animal trials have been conducted in this field, few clinical trials have so far followed in which ES therapy was translated to humans [31, 34, 61, 69, 70, 71, 72, 73].

## 4 Artificial Reinnervation to Enable Functional IMS

Instead of direct stimulation of denervated muscle, an alternative strategy is to attempt to reinnervate the muscle, and then apply stimulation to the reinnervating nerve cells. If this can be achieved, then in principle the techniques of conventional FES, as used for UMN injuries, can be extended to LMN injuries. This in theory would avoid many of the disadvantages of DMS including discomfort, high power levels, uneven muscle activation, and the potential for tissue damage.

### 4.1 Reinnervation of Muscles In Situ

Muscle reinnervation is one of the most important obstacles to overcome in order to achieve functional IMS rather than using DMS for denervated muscle. Baxendale and Andrews [74] reported their initial attempt in 1992 to reinnervate denervated muscle by directly implanting neonatal neuronal grafts into the denervated muscle of rats. Their results demonstrated neuronal survival within the host muscle for 12 weeks. After week 12, the experiment terminated, and it remains possible that neuronal survival persisted beyond the 12‐week timeframe. Around the same period, Erb et al. [75] tested whether reinnervation could be achieved by using embryonic motor neurons transplanted into axotomised nerves. Embryonic rat ventral horn cells transplanted into the distal stump of the transected tibial nerve in adult rats survived in this environment and were able to reinnervate the gastrocnemius muscle. Grumbles et al. [76] subsequently explored the timing of cell transplantation in relation to injury, showing that pre‐degeneration (making a prior nerve injury so that Wallerian degeneration is underway before the time of transplantation) enhanced axonal growth. The same group went on to demonstrate that ES of transplanted motor neurons improved motor neuron survival, axon regeneration, muscle reinnervation, and muscle function [77, 78, 79]. Kurimoto et al. [80] demonstrated the combination of neural stem cell transplantation with FES in a rat sciatic nerve transection model, showcasing the potential of this integrated therapeutic strategy to restore functional muscle activity. Tokutake et al. [81] further advanced this approach by developing a dedicated wirelessly powered stimulator system.

Most research to date has used embryonic spinal cord tissue as a source of motor neuron stem cells. Translation of this work to humans would raise major ethical barriers. Fortunately, we now have techniques to generate motor neurons from non‐embryonic sources, using autologous induced pluripotent stem cells (iPSCs) that are then differentiated down a motor neuronal path [82]. This is likely to mean that the use of embryonic tissue in human studies is not required. Andrews and Ray [83] proposed the use of neurons derived from adult mesenchymal stem cells for reinnervating muscle in FES, offering a promising alternative that circumvents the ethical concerns associated with embryonic or neonatal human cells.

Another area where the animal models differ from the intended clinical application is the chronicity of the injury. In animal studies, cells are transplanted either to a freshly cut injury site or to a site where there has been a short period of Wallerian degeneration, and the acute setting together with short regeneration distances in rodents are conducive to good reinnervation of the target muscle. By contrast, in clinical practice, most patients have experienced long‐term muscle denervation. With longer periods of denervation, even with good axonal regeneration from a proximal nerve stump and anatomically normal looking neuromuscular junction (NMJ) formation, synaptic activity may not be well reestablished, and functional NMJ recovery is severely compromised. This is seen in rodents after denervation periods as short as 1 month [84]. In humans, functional recovery is affected strongly after 12–18 months of denervation [85]. More research on the functional recovery of long‐term denervated muscle and the best time window of reinnervation acceptance are necessary before clinical translation is likely to be successfully realized in the usual chronic injury setting.

### 4.2 Reinnervation of Transferred Muscles

In some clinical situations, muscles may be transferred surgically to ectopic sites in order to fulfill a new functional role. An example is the latissimus dorsi muscle, which has been transferred from the back through the chest and used to augment cardiac function in chronic heart failure. This may be achieved either by wrapping a muscle flap around the heart itself and stimulating it with an implantable cardiomyostimulator to reinforce cardiac systole (dynamic cardiomyoplasty) [86] or by wrapping it around the thoracic aorta and stimulating the muscle to provide counterpulsation in diastole [87].

Muscle transfer has other potential applications, including treatment of fecal and urinary incontinence by wrapping transferred skeletal muscles around the target site to form a new sphincter. However, the possibilities for other applications are limited by the need to preserve innervation to permit stimulation, generally restricting movement of muscles to within the distance permitted by a neurovascular pedicle. In a few cases, notably complex procedures to restore function after brachial plexus injury, free muscle transfers have been reinnervated with nerves taken from another muscle [88, 89]. An electively transferred muscle, freshly denervated, should be maximally conducive to recovery of normal NMJ function. This requires a source of a suitable transferrable nerve, which is within range of the target, and where denervation of the “donor” muscle does not cause unacceptable sacrifice of function. If the transferred nerve does not reach all the way to the target, a nerve graft may be required to bridge the gap, with further sacrifice of function elsewhere and potentially lengthy reinnervation times.

These problems could be diminished with reinnervation of transferred muscle by a depot of active nerve cells maintained nearby. The muscle would become excitable via FES type activation of the nerve cells in the depot. Compared to transfer on a neurovascular pedicle, cell‐based reinnervation would allow complete freedom of the muscle donor site relative to the destination site. Compared to reinnervation by nerve transfer, this approach could offer several theoretical advantages, such as the ability to position the nerve source close to the target muscle, which could potentially shorten reinnervation time while avoiding loss of function in the donor nerve. It would also eliminate the anatomical limitations of pedicled transfers. However, the practical implementation of such cell‐based reinnervation strategies remains complex and is still largely experimental.

## 5 Conclusion

Numerous studies have shown the benefits of ES of denervated muscle in terms of maintenance of muscle mass and some potential for restoration of function. Presently the preservation of muscle mass and prevention of muscle wasting (cushioning) are the major contributions of ES. Functional restoration of daily movements in denervated muscle is less well demonstrated in comparison. The practical difficulties of DMS have largely prevented its adoption, in stark contrast to the widespread use of FES for innervated muscle.

The thousandfold difference in energy requirements to stimulate denervated muscle compared to innervated muscle is the central problem. Or put conversely, the remarkable efficiency of normal neuromuscular activation is difficult to achieve by other means. Issues including discomfort, the potential for tissue damage, and the necessity of large electrodes limiting specificity all stem from the very high stimulus intensity required for DMS. The landmark project RISE is now two decades old, and a solution for these problems is still needed. A new method that can reliably, controllably, and selectively stimulate denervated muscle to obtain functional restoration is greatly needed.

We believe that the approach of combining the reinnervation and indirect stimulation of reinnervated muscle offers that solution. Artificial reinnervation will permit the use of more standard, low energy FES techniques, as are applied to patients with upper motor neuron injuries and muscles that are paralyzed but not denervated. This will represent a quantum improvement on existing techniques for DMS. Moreover, the novel use of stimulation in free muscle transfers may open up entirely new treatment possibilities.

## Author Contributions

L.C. and J.J.F. conceptualised the article. L.C. prepared the original draft. All authors provided critical review and editing of the manuscript.

## Conflicts of Interest

The authors declare no conflicts of interest.

## Data Availability

Data sharing not applicable to this article as no datasets were generated or analyzed during this study.
